# The coughing body: etiquettes, techniques, sonographies and spaces

**DOI:** 10.1057/s41292-020-00196-3

**Published:** 2020-09-15

**Authors:** Nik Brown, Sarah Nettleton, Chrissy Buse, Alan Lewis, Daryl Martin

**Affiliations:** 1 Department of Sociology, University of York, York, YO10 5DD UK; 2 Manchester School of Architecture, University of Manchester, Manchester, M15 6BR UK

**Keywords:** Respiratory infections, Social distancing, Coughing etiquettes, Body boundaries

## Abstract

With newfound relevance in the context of Covid-19, we focus on the coughing body, building on an in-depth qualitative study of three UK lung infection clinics treating people with cystic fibrosis. Conceptually we take our cue from Norbert Elias and the way something as physiologically fundamental as coughing becomes the focus of etiquette and technique, touching also on themes central to Mary Douglas’ anthropology of pollution. This is explored through four themes. First, we show how coughing becomes a matter of biopolitical citizenship expressed through etiquettes that also displace pollution anxieties to surroundings. Second, coughing is a question of being assisted to cough through the mediation of professional skills, interventions and devices. Third, coughing is seen to be central to the sonographic soundscape of the healthcare environment whereby people learn to recognise (and sometimes misrecognise) each other through the ‘sound’ of the cough. Finally, coughing properly can be seen to have both a ‘time and a place’ including the retreat of the cough from public space into risky confined spaces. Our conclusion speculates on the way these insights shed light on aspects of life that, until the Covid-19 pandemic, lay largely hidden.

## Introduction

With particular significance in the context of the Covid-19 pandemic, this paper focuses on what we call ‘the coughing body’ and how it is that the cough becomes a central organising social and embodied feature of life for people with respiratory illnesses. With some exceptions (Leslie 2006; Lowton 2004; Lawlor 2006; Parish 2011; Hansson 2019; Bailey 2008), there is remarkably little research on coughing in literature related to the sociology and anthropology of the body. We take our initial cue from Elias ([1939] 1994, pp. 117–125) whereby something physiologically fundamental becomes a matter of proper etiquette and bodily technique (Mauss 1973), an unavoidable physiological imperative that must however be acquired, learnt and even taught. Coughing also touches on themes central to Douglas’ ([1966] 2003) anthropology of pollution, transgression and the ritualised restoration of bodily boundaries.

Covid-19 ushers in a historical moment that attaches new and troubling meaning to coughs and coughing. This paper reports on a UK-funded qualitative research project entitled *Pathways, practices and architectures: containing antimicrobial resistance (AMR) in the cystic fibrosis clinic* (Brown et al.^ 2019^). Here we explore themes of contagion in hospital respiratory clinics treating people with cystic fibrosis (CF), a genetic condition characterised by multiple co-morbidities, including chronic life-shortening respiratory infections. From around the early 1990s, it was recognised that interpersonal contact and physical interaction between people with CF had resulted in cross-infection leading to epidemic respiratory infections amongst the global CF population (Conway 2008; Ashish et al. 2013). Since that time, reducing transmission has come to depend on practices that have now become familiar to everyday life in a pandemic. This has included ‘segregation’ and ‘physical distancing’ from others with the condition, but also ‘self-isolation’ during flu season, and ‘shielding’ to protect oneself or other household occupants from potentially dangerous infections. Indeed, the ‘ six foot rule’ or ‘two metre rule’ was first coined in the context of CF becoming, as we will show, a deeply embodied feature of life for those prone to life-threatening lung infections (CF Foundation 2014).

The paper explores this with respect to four interconnected dimensions of coughing in the lives of people living with chronic respiratory infections. First, we show how coughing becomes a matter of responsible ‘biopolitical citizenship’ (Rose and Novas 2004) physically and materially expressed through the etiquettes of coughing. And yet, we are also interested in how this displaces pollution anxieties to surrounding objects, surfaces and people. Second, coughing is, in the context of CF, also a matter of being assisted to cough ‘properly’ by trained professionals, often technically mediated with the further assistance of devices used to ‘produce’ fluid from the lungs. Third, coughing is central to the sonographic soundscape of the healthcare environment whereby people with CF learn to recognise (and sometimes misrecognise) each other through the ‘sound’ of the cough. Finally, coughing properly can be seen to have both a ‘time and a place’, a carefully orchestrated occasion having also a proper spatial locus.

In the context of a respiratory pandemic, the sounds, sights and experience of coughs and coughing have become part of a public scenography with far-reaching social and biopolitical implications. Our own work with the CF community recalls multiple moments in which coughs attract wider public attention. For instance, in October 2017, the former British Prime Minister, Theresa May’s premiership hung precariously in the balance as she launched into a conference speech that might, or possibly not, save her premiership. But her emphatic claims about the rude health of the British economy crumpled into a merciless hacking cough that steadily worsened as she painfully persisted through her speech. The following day’s headlines were ruthless in perpetuating the deeply gendered media focus on May’s apparent lack of feminine warmth (‘ice queen’, ‘frosty May’, ‘Maybot’) breached by an involuntary eruption of uncontrolled coughing. The calamitous episode is worth recounting here because of the way it touches on themes central to this paper. Coughs are precariously suspended between the voluntary and the involuntary, chaotically spontaneous and yet subject to cultured techniques of etiquette and self-management. Coughs are sonographic events, disruptive outbursts of noise, but also bearing meaning. Without question, a cough has a proper time and place.

We conceptually locate the coughing body in literatures on the socio-materialities of etiquettes, pollution and hygiene. Elias’s essay ([1939] 1994) “On blowing one’s nose” charts the sociogenesis of an embodied etiquette of shame and revulsion applied to the nose. Early modern texts admonish those tempted to “blow one’s nose with the same hand that you use to hold the meat” (117). Somewhat later, the use of the handkerchief in the sixteenth century is accompanied by the instruction not to “spread out your handkerchief and peer into it as if pearls and rubies might have fallen out of your head” (119). Discussion of the nose and its secretions and discharges becomes unmentionable, an expanding “shame frontier” of decency that forbids what had previously been possible in the company of others, but which now must be done in private, sequestrated and out of plain sight.

Unavoidably natural imperatives like defecating, menstruating, urinating, sleeping, become fenced off from the public scenography. Dishonour attaches to those whose lack of individual control is expressed in nasal hygienic ill-discipline. For Elias, the seemingly trivial etiquettes of the nose are part of the fashioning of the modern sovereign subject, the bounded bodily self, persons discretely separable from one another. The open and incomplete body becomes enclosed, isolatable, an intimate and private thing, a self-possession that is both internalised whilst also expressing to others one’s prestige. One maintains the integrity of the self, and one’s place in the world, by avoiding those whose bodily boundaries communicate disintegration and leakage.

Coughing is, as we will see, clearly divided between foundational categories of purity and danger (Douglas [1966] 2003), between the sacred and profane, symbolic-material categories that designate spaces and places of embodied belonging and non-belonging. Importantly, Douglas argued against simplistically medical materialist and sanitary explanations for the category of dirt. Kristeva echoes this in writing that “… it is thus not lack of cleanliness or health that causes abjection but what disturbs identity, system, order” (1982, p. 69). But this doesn’t deny the embodied viscerality of pollution. As Kristeva reminds us, pollution is an occasion for jettisoning that which opposes, an occasion that brings on retching, vomiting and nausea. Douglas writes of medical materialism in a much more “extended sense” as the justification of ritual practices in “terms of aches and pains which would afflict [us] should the rites be neglected” ([1966] 2003, p. 40). She also “deplores” the attribution of symbolic ritual to the “primitives” and clinical hygiene to the “moderns”, denouncing the idea that “our washing, scrubbing, isolating and disinfecting has only a superficial resemblance with ritual purification… Our practices are solidly based on hygiene, theirs are symbolic. We kill germs, they kill off spirits” (ibid). All biomedicine is therefore symbolically charged and deeply mythic.

Coughing is a matter of the specific spatialisation of the body ‘within’ particular spheres, or ‘sphereologies’ of association and disassociation (Sloterdijk 1998, 2004; Brown 2018), the expression of hygiene and bodily order through successive historical ‘political geometries’ (Armstrong 1993; see also Wakefield-Rann et al. 2019). For Armstrong, the first such geometry is located in quarantine separating the sick from the healthy, to be locked in time and place for the duration of the threat. Arrested movement depends more upon the classification of risky places. The subsequent shift from quarantine to sanitary science sustained the emphasis on place but now elaborated with reference to the environment of the body (soil, climate, air, sunlight). Around the turn of the nineteenth and twentieth centuries, sanitary science is succeeded by the regime of ‘personal hygiene’, the active self-monitoring of one’s own bodily boundaries. Where sanitary science was concerned with the population, personal hygiene brings into focus ‘countless individualities’, their habits, conduct and pre-eminently their ‘behaviours’ or ‘idiosyncrasies’. The hygienic regime fixes its gaze upon the interpenetration of bodies, the generally negative nature of intercorporeal transmission. Once a disease of one’s condition (poverty, poor sanitation), the new dangers emanated from the “bodies of others, from contact with a tuberculous patient who was coughing or spitting” (Armstrong 1993, p. 404). Each of these geometries have come into play, in one way or another, both in our own more focussed study and now in the wider context of a pandemic emergency. However, as we go on to show below, both CF and Covid-19 unquestionably focus attention on the immediate atmospheric environment of the coughing body, its ‘halo of air’ (Brown et al. 2020), and its containment.

## The study

This paper develops insights from a 2-year study undertaken within three cystic fibrosis hospital-based clinics, concluding in January 2020, thus immediately preceding the Covid-19 pandemic. Our research included ethnographic observation, face-to-face and walking interviews with 54 respondents (34 hospital staff, 15 patients, 2 family members and 3 architects). Architectural layout plans of the clinics were used in face-to-face graphic interviews with participants encouraged to annotate plans, using different colour markers indicating walking routes, cross-infection ‘hot spots’, design features, etc. We conducted 70 interviews, 45 graphic and 25 ‘walking interviews’ with participants guiding us through hospital buildings, taking photographs of spaces, objects, signage and using the built environment as a prompt for discussion. Walking interviews followed routine journeys through the hospital space (including entrances, exits, corridors, treatment rooms, waiting areas, wards, etc.). Ethnographic observations were undertaken in CF outpatient clinics and inpatient wards with facilities for ‘ad hoc’ outpatient appointments. Incidental observations were conducted during visits to each site over a 9–10-month period, in addition to 72 hours of ‘targeted ethnography’ (Sage and Dainty 2012) involving focused observations of clinics during those days on which people with CF were being treated in hospital. Our project also involved archival and historical research into hospital building design (see also Prior 1992; Brown et al. 2020), including patent applications related to respiratory devices and infection control equipment.

All data, both textual and visual materials, were analysed using qualitative analytical software (NVivo) resulting in the coding of data against a wide range of themes and subthemes. This has enabled analytical links to be made across different types of textual and non-textual data. The present paper is the outcome of a number of more focussed codes and sub-codes connected to coughs and coughing. The related data were further analysed and discussed in a series of analytical sessions undertaken by the authors. Ethical approval for our research was secured through the UK NHS and the University of York UK. Names of all participants and participating hospitals have been replaced with pseudonyms.

## Coughing techniques and etiquettes

Coughs come and go, but for most people with CF, the sensory irritation of ‘cough receptors’ in the epithelial lining of the respiratory track is continual and constant. To cough can therefore become a moment by moment involuntary and uncontrollable necessity without which one would drown in mucous. “With CF”, one patient explains, “it can just hit you out of nowhere, so you might not even have time to like cover your mouth and things like that” (Abbi—patient). Indeed, spending time with CF people in the course of our research has been a question of often pausing for coughing to occur, of briefly suspending the conversation, allowing time for ‘composure’ to return and the interview to resume. There’s a mutual and carefully observed choreography to these momentary intervals, politely ritualised adjustments of posture, sips of water, the use of tissues. The cough may be directed away and down towards the floor. There may be a sensitively handled exchange of apologies and expressions of concern. The interviewer may sit back slightly to signal an intermission, to ‘take a breather’. Bouts of involuntary coughing are reminders of what it is that we are there to explore together.

The embodied repair of order (Goffman 1969) interrupted by coughing takes many forms. Corrective strategies focus particularly on the hand and the use of the hand (the back of the hand, the cupped palm, the wrist, the clenched fist) to inhibit or restrict the expulsion of mucosal droplets into the surrounding air. A senior CF doctor (Nathan) demonstrated to us the cupping of the hand and turning the head away from those who maybe nearby, especially he said, when passing on corridors. Abbi (patient) described how she would attempt to protect “the general area” by “trying to cough into my arm… or into a tissue or into a jumper if I’ve got a jumper, just to protect other people really”. The back of the wrist, forearm and the crook of the elbow feature prominently in these discussions on coughing. In the context of Covid-19, to cough into the elbow rather than the hand has since become ubiquitously reflected in public health advice on ‘coughing etiquette’ (CDC 2007).

Coughing clearly threatens a cluster of distinctions sacred to the biomedicalised subject, a reflex that is at once involuntary and yet required to conform to rules of place, privacy and self-containment. A cough confounds demarcations between control, reason and rationality on the one hand, and on the other, unthinking involuntary spontaneity. Coughing is both a source of symbolic pollution whilst also threatening contagion and the airborne spread of infection emitted from the most visible of our orifices, the mouth (see Nettleton 1988). Coughs erupt from the bounded body becoming aerosolised in the surrounding environment, a bodily action that is required to conform to, and yet troubles, rules of self-governance. Coughing ‘properly’ is embedded in power relations that have an “immediate hold upon” the body, that “…invest it, mark it, train it, force it to carry out tasks, to perform ceremonies, to emit signs” (Foucault 1991, p. 25).

Being directed to cough ‘into’ or ‘onto’ something other than the air both resolves and yet displaces pollution anxieties to the immediate atmosphere of the body, to other surfaces, objects, clothing, fabric, hands, materials and handkerchiefs. A patent application for a “cough catcher” (US 2014/0325738A1), an absorbent fingerless glove to be worn on the hand, makes the point that “tissues are not always readily available when one needs to cough” (1). The text notes that cough-catching techniques may commonly include the use of tissues, “one’s upper arm, elbow, or shoulder”. Whilst all are better than using the hands, they nevertheless risk the later transfer of pathogens “conveyed to others”. The cough catcher aims to resolve the arbitrary unpredictability of a sudden “fit of coughing” with a hand-worn disinfectant-infused glove covering the palm and top of the hand. “The method may further involve removing the apparatus from the hand of the individual after the cough”, it continues, “and disposing of the apparatus after removing” (ibid). Other patents are filed for single-use disposable wearables including a “cough sleeve” (US 2012/0066816 A1), a “cough suppressant garment” to be worn around the neck (US 2013/0198934 A1), the “cough cuff” (US 2011/0088132 A1) worn at the elbow, the latter citing US Centers for Disease Control and Prevention (CDC) recommendations that people “bend their arms and cough into the bend of the elbow, not into their hands”. A patent application for a “cough germ containment device” (US 2009/0270831 A1) goes a step further enabling the user to cough into a cylindrical tube fitted with a “gas-permeable anti-bacterial filter”. The design includes a lanyard to attach the device to the neck. We take up the question of sound and the sonography of coughing further into this paper, but another application for a “cough muffler” incorporates an “absorbing mass” designed to “deaden the involuntary sounds of coughing… in a manner as to be non-disturbing to adjacent or surrounding” persons on those occasions that may require quiet (US-690859864).

So much therefore depends on the materialities, affordances and techniques by which a cough comes to be contained, or not, or passed on, transferred and channelled from the respiratory track to the mouth, to the air, or to other parts of the body and to fabrics and surround surfaces (see also Mesman 2012). People with CF are advised not to swallow sputum expelled from the lungs during coughing but instead to spit into a disposable handkerchief or preferably a sealable ‘sputum pot’. Concerns have come to focus not just on the colonisation of the lungs by pathogens, but also that of swallowed mucous, the intestinal tract and faecal matter resulting in environmental contamination. Isobel, a patient in her early 50s, found herself often conflicted about spitting or swallowing her sputum:

… because it comes into your mouth, and it’s, you can’t do it there because people look, and the only alternative the lady gave me was going to the toilets, but then you’ve got to get up and move, and it’s in your mouth, and you can’t go, so I swallow it, because it’s more embarrassing to bring it out, which is not what you’re supposed to do (Isobel - patient).

Continual coughing can constitute the stigmatising production of unacceptable matters, “abominations of the body” as Goffman (1963, p. 4) once put it, an expression that closely anticipates Douglas’ own account of pollution. Isobel felt self-conscious when in the hospital’s general waiting area, sometimes aware of others “tutting”, or “really upset” at her coughs. “They physically move away”, she says, “because you’re coughing all the time, and they’re worried, fair enough… They don’t know they can’t catch it, but they don’t want to”. Echoing Elias’ (1939) point about the retreat of bodily processes from communal space, Isobel felt ashamed and humiliated at these sudden respiratory eruptions and the prospect of expectorating sputum from the mouth publicly (see also Turner 2003 and Shildrick 2015).

## The assisted cough

To cough is as fundamental to life as breathing itself, a reactive ‘reflex’ without which our ‘airways’ would become flooded with pathogens and particulates. Coughing properly in the context of CF is also a matter of being ‘induced’ to ‘loosen’ the unusually thick respiratory mucosal lining of the lungs. This routinely involves the assistance of physiotherapists trained in the art of the cough and the use of ‘cough assist’ machines, ‘flutter devices’ and nebulised treatments. Måseide (2011) has similarly traced the ‘body work’ (Twigg et al. 2011) and techniques (Mauss 1973) by which respiratory examinations are accomplished through communicative instructions, technical mediation, and the cooperation of the body under examination.

In the context of CF, coughing is crucially a means of obtaining sputum for bacterial diagnosis. Patients are then classified and spatio-temporally ‘cohorted’ according to their infections (resistant and non-resistant, epidemic and non-epidemic, etc.). The identities of patients and their navigation of space (see below) become intimately entangled with the strain of infection for which they test positive (see also Wakefield-Rann et al. 2019). The whole patient journey commences with a bacterial diagnosis that comes to depend on coughing correctly to produce a ‘good sample’. Both physiotherapists and patients are under some degree of pressure since coughing properly can have serious implications for segregation and safely preserving the distance between patients who should not mix. Coughing underpins what Robinson (2019) has called “microbial performativity” whereby the ecology of pathogens shapes socio-technical life. However, “your classification”, we were told by one physiotherapist, is “only as good as your last sample” (Anne—physiotherapist). Coughs become the focus of constant evaluation with patients classified as either “pretty good” or “bad” coughers. Not coughing properly, and not producing sputum, can result in having to resort instead to a “cough swab… which is less accurate” (Ellie—consultant). Another physician explains:

… sputum will identify all infections… cough swabs… miss nearly everything… [we have an] adult who’s 19 years old and in the last five years he’s never produced sputum, just a cough swab, and he’s not doing very well, so … we’ll do a bronchoscopy, put a camera down… we wash out the lungs, and we’ll find *cepacia*, *mycobacterium abscessus* and all these other things (Luke – consultant).

Coughing on command therefore doesn’t necessarily come easily even in a condition like CF characterised by constant coughing. Some older patients may have more experience at ‘producing’ fluids from the lungs, the result of acquired skill or simply being more congested. Patients come to self-identify themselves as good or bad at their ‘sputums’. One younger male patient (Rupert) said “I’m awful at sputum… if by a huge chance I bring up sputum, it will be me running or playing rugby… I will treat it like a gem until I come in… but I’m not a good CF patient … I won’t give a sputum, never bring anything up”. That fact that sputum can become ‘gemlike’ in this way is connotative of the complex symbolic nexus within which mucosal fluids are nested.

We’ve already noted above the embarrassment that attaches to the act of expectorating. But even when attended in the privacy of a treatment room by clinical staff, the possibility of shame can persist. Patients talk of finding it socially ‘uncomfortable’ to produce sputum in the company of clinicians, or sometimes just ‘too hard’. Luke (consultant) describes the problem they have with younger patients, “especially young girls [laughs] will say… oh, I never produce sputum”. But, he continues, “you can hear it rattling around in their chest… sometimes they genuinely have a very dry chest, then sometimes it’s a psychosocial thing of not…. being happy to cough it up… not wanting to”. Again, here we find the biomedically informed mandate to expectorate, even when part of a highly ritualised clinical routine, in tension with symbolically charged and gendered categories of shame and humiliation (see also Chapple et al. 2004). This is therefore a pollution problematic marked in multifarious ways including gender, age and place.

Inevitably, attending so closely to those who cough transfers pollution from the person coughing to those assisting them. Whilst largely harmless for staff, this potentially puts other CF patients at risk of transmission. To officiate in such close proximity is a source of concern for clinical staff who may be continually touching potentially contaminated bodies, surfaces, objects, and moving between patients who themselves remain separated:

… to really maximize that production of sputum… we’re in the room with them when they’re nebulising… carrying out exercise and airway clearance. … we are potentially more of a risk in terms of cross-infection… we are … in the room when they’re doing the high risk activities… encouraging them to cough, to clear … improve their ventilation, we’re using pieces of equipment … so potentially become a risk for someone else (Rachel – physiotherapist).

Practitioners come to envision themselves as vectors of transmission: “If someone coughs… at what point are you not covered in *pseudomonas* [bacteria]… would be nice to know” (Mandy—physiotherapist). Prevention of cross-infection is expressed through a demanding array of sanitary and symbolic practices, unsurprisingly those concerned with hand hygiene (gloves, hand washing, sanitiser), but also constantly having to clean equipment and especially door handles. “They’re dirty things, door handles”, explains Irene, a physiotherapist, “they’ve been coughing… they touch the door handle, that’s an obvious point of transfer”. Between patients and staff, a loosely defined division of hygienic labour separates the use of hand sanitiser from water-soap hand washing. CF patients spoke about how they sometimes felt a lack of ownership over taps, sinks and soap implicitly regarded as belonging to clinical staff (see also Pink et al. 2014).

Wearing protective clothing varies greatly between clinics, practitioners and different kinds of practice. “We don’t tend to wear aprons or gloves”, Mandy (physiotherapist) told us, “we just wash our hands”. Ellie (clinician) demonstrated putting on a plastic apron and then, to illustrate, pointed to all the parts of her body and clothing left uncovered. Rachel (physiotherapist) explained that whilst guidance advises the use of protective clothing, her “gut feeling is that aprons are fairly useless… when a patient’s coughing, its going everywhere… there’s just not enough strong evidence”. Ivor, a hospital microbiologist, also pointed to the divergence between evidence, guidance and practice. Aprons are being worn to visibly symbolise alignment with guidance, in the absence of ‘robust’ research, and without the wearer necessarily believing it to be materially effective:

… the guidance … is to wear aprons, gloves, possibly masks as well… if you’ve got somebody who’s coughing… and there’s little globulets of sputum coming out, and they hit the plastic apron, which you can take off and put in a waste bin…that’s the theory … looking for robust evidence that you break the chain of transmission definitively by wearing an apron versus you don’t by not wearing it, is difficult to come by (Ivor).

All of the coughing etiquettes discussed above inevitably produce a surplus of deeply problematic material wastes, an excess of leftover aprons, gloves, hand towels, sputum pots and tissues, etc. to be handled and discarded responsibly. But the ‘excess’ produced by coughing isn’t only material, it is also sonographic.

## Sonographies of the coughing body

Healthcare spaces are intensely acoustic environments that prompt a new alertness to the implications of ‘noise pollution’ for occupational health and patient wellbeing. The hospitals in our study reverberate with the cacophonous clamour of sirens, TV monitors, alerts and alarms, ringtones, announcements and notifications, the noise of cleaning, urban traffic, trolleys and furniture in motion, voices, chatter, and sometimes the sounds of discomfort and pain (Rice 2003, 2013). In particular, coughs and coughing puncture healthcare soundscapes, rising above ‘background noise’ and becoming, in the context of respiratory care, overt and explicit objects of concern. Indeed, coughing on the wards and around treatment rooms can be “relentless” (fieldnotes Summer 2019). Coughs locate bodies in space and serve as diagnostic markers, and semantic cues with which to recognise, and sometimes misrecognise, others with cystic fibrosis.

With respect to questions about infectious disease, contagion and pollution, there is something poignant in the way that sound constitutes a form of ‘touch’, direct contact, or physical interaction at a distance. Cobussen writes of the way noise has the capacity to “contaminate” such that it “affects and infects any system, any order” (2005, p. 30). As Gunaratnam (2009) puts it in the context of the palliative care soundscape, “… to hear is to be literally touched and to take impressions of others into our bodies whether we like it or not” (5). The material force of the cough physically moves the air, travelling through space, entering and penetrating the body of the listener, oscillating and reverberating auditory tissues and nerve fibres. Coughing is ‘sono-material’. For sure, the cough is a ‘commotion’ in as much as it represents ‘noise’. But coughs are also ‘co-motions’ between bodies. They are directly physical, interpenetrative and intercorporeal in this sense. The cough collapses the distance between bodies; there is a slippage between auditory, acoustic and infective contagion at play in the sound of coughing.

For people with CF and those caring for them, the cough has become a mode of ‘auditory work’, of ‘learning to listen’ to the CF body and the location of the body in space (see also Maslen 2016). One CF healthcare worker (Mary) told us that, whilst waiting for patients to arrive, “you’ve always got to be keeping an ear open really… If I hear cough I’m like…! [looks around her]”. Such sonographic labour is a belatedly ‘under-examined’ dimension of scholarship on embodiment, let alone breath, breathing and respiratory life (Allen-Collinson and Owton 2012; Robinson 2019). In particular, such listening is a matter of being able to recognise, or not, other people with CF. For some of our participants, coughs represent a form of insider group recognition, a secret code amongst those for whom coughing is a vernacular dialect, a non-verbal idiom. In an interview with Tina (patient), she talked of the way people with CF will often have “salty skin”, a characteristic of the CF condition resulting in unusually high levels of sodium excreted in sweat. But you can’t simply “lick” someone’s skin to see if they’re CF or not. Instead, the “one tell-tale sign” is the “constant cough… a bit like mine… [they] could possibly have CF”.

Rupert (patient) spoke at length of the way people with CF will often “look pretty normal” but how you can tentatively “kind of tell by the kind of cough people have… if it’s a proper phlegmy cough, that’s classic CF … almost sounds like a smoker’s cough”. Laura (patient) recounted how she has “a couple of friends who smoke… you can hear it”, but they “don’t have that mucusy sound with it, it’s just a deep cough”. Isobel (patient) too was keen to differentiate between the CF and smoker’s cough and was anxious about being mis-identified by others as herself a smoker. Laura also spoke about being able to identify the sound of an infection cough. “If I hear somebody’s cough”, she explained, “I know whether they’re carrying an infection… that’s an infection cough… So I can recognise coughs, but I wouldn’t say that’s a CF person”.

For people with CF, coughs are therefore ‘sonorous expressions’ (Vannini et al. 2010, p. 331) integral to safely navigating the clinical environment. Whether a cough is that of someone with CF, or that of a smoker, or different kinds of infection is a learnt and embodied auditory attunement to the respiratory tones, nuances and cues of oneself and others. Laura used the word ‘ruckle’, a colloquial idiom suggesting a hoarse rattle, to distinguish the infected CF cough from that of a ‘dry’ viral infection:

It’s the sound. When you’ve got an infection you’re very congested, and you can, you can hear the phlegm. And to me, if they’ve got phlegm they’ve got an infection. And it’s a sound that I’ve lived with that long, that you just know it… I know if it’s a cold cough it’s viral, because it’s just different, the sound’s different from a cold cough to an infection cough. … It’s dry…. and it’s a different cough to someone who’s carrying congested mucus on the chest… When they cough it’s… just a deeper, deeper sound, and it comes with a ruckle after it, like they need to clear, and they need a good cough to clear.

Deciphering coughs however is an uncertain business involving the inexact interpretation of visual and auditory cues. As another patient put it, “it’s the CF cough. Sometimes you get it wrong, it’s somebody with just a bad cough, but it is just a very distinctive cough” (Indigo). The sound of a cough becomes a trigger for more careful visual scrutiny including whether “… someone’s got their port bandaged up or… they have an inpatient band and they’re coughing … chances are… they’ve got CF” (Alison). If “anybody’s coughing”, Amy would “look at their hands to see what the shape of the fingernails are like”, searching for evidence of “finger clubbing”, a swelling of the fingertips resulting from low blood-oxygen levels and a common symptom of poor pulmonary function. Learning to listen, and to see, in this way is a finely grained corporeal attunement to the sonography of breath, an auditory episteme of ‘acoustic knowing’ (Feld 1996) and ‘deep listening’ (Bull and Back 2003, p. 3).

It is in this sense that Rice (2003, 2013) writes of “sounding bodies” and the “body as a soundscape”, as “aurally perceptible”, and the importance of listening through the practice of diagnostic auscultation, the role of auditory devices, especially the stethoscope. Just as people with CF use the cough as the auditory prompt for further scrutiny of the bodies of others, Rice documents the way hospital soundscapes articulate with “visual surveillance”, extending the “panoptic possibility” of a “sonically ordered sense of self” (2003, p. 8) in which patients are able to spatially situate themselves.

## Spatialising the coughing body

Coughs define the geometries of the private and the public, the individual and the communal, the sacred and the profane. They give biopolitical meaning to waiting rooms, corridors, lobby areas and treatment rooms. That meaning extends to the invisible, the shape of air and what is ‘carried on the air’. Coughs perforate the distance separating one body from that of another. Such moments are reflex triggers for spatial repositioning, re-orientations within one’s immediate surroundings. Rupert described his acute awareness of “… anybody coughing, sneezing, or anybody who looks unwell… I sort of take four steps across… or get a tissue and cover my mouth”. Respiratory vigilance is “as simple as looking both ways when you cross the road”, he said.

All too familiar now in life under Covid-19, Rupert went on to describe how he would hold his breath at moments where he perceived respiratory threat, a deeply symbolic means of closing off the borders of the body, an anonymous and unobservable form of withdrawal. It is also a form of waiting (‘don’t hold your breath’), an inevitably time-limited pause allowing a threat to pass:

… if I was in a room and there was someone coughing.. I would want to get out of the room. … if I’m on a train or something … someone is coughing, I will hold my breath… The same with smokers as well, I hold my breath around them…. if there’s people coughing… you shut your mouth … if I know somebody with CF’s walked past, and they’ve coughed, I’ll hold my breath! For as long as I can. I do that in the supermarket.

Rupert spoke of discovering that his university rugby coach also has CF. “If he’s got a cough”, he said, “…I’m going to maybe stay away from the circle and not do team talks”. The ‘circle’ and the ‘scrum’ in rugby both constitute extreme moments of intimate physical contact in which ‘viscoelastic bodies’ (Newman et al. 2016) merge. For Rupert, rugby is something of a metaphor for his precarious navigation of biopoliticised space. Whatever measures you take, he says, “you’re still breathing the same air”. Whilst at university “… for all I know I could have been in multiple random classrooms with people with CF for years, and not knowing it”. He went on, “you can’t put yourself in a bubble, can you? You’ve got to live”. In this sense, bubbles of self-isolation both preserve and yet also endanger life. Similarly, in an auto-ethnography of asthma, Hansson (2019) writes of ‘critical places’ where physical risks are seen to be in tension with social benefits.

The coughing body is routinely envisioned as a spatialised atmosphere in both lay and biomedical discourse. Some microbiology literature describes coughing bodies meteorologically as ‘clouds’ (Sherertz et al. 2001). Here, clouds recast the body as a spatially transgressive bioaerosol echoing more recent debates and uncertainties on air as a medium of coronavirus transmission (Lewis 2020; Bourouiba 2020). The borders of the coughing body are not delimited by the skin but by its ‘atmospheric halo’ (Brown et al. 2020).

Already noted above, the US-based CF Foundation (2014) recommends a minimal distance of six feet ‘6ft rule’ be observed between people with CF. Meticulous spatial observances of this kind are common amongst participants in our study. Tina, for example, was particularly anxious about hospital waiting areas. She would choose a seat as far from others as possible and secure the area by spreading her personal belongings on adjacent chairs to preserve what she called her own ‘personal bubble’. For her, the bubble is a delineated volume within which to cough without drawing unwelcome attention, and to be protected from the coughs of others. The ‘bubble’ is a deeply spherological conception of biopolitics (Sloterdijk 1998, 2004) having now taken on newly globalised significance in the Covid-19 crisis (see Rankin 2020). Echoing many themes central to Douglas and Elias, Sloterdijk diagnoses in the contemporary period a ‘bubbling-up’ of ‘co-isolated adjacencies’. Pandemic life can so suitably be expressed as an aggregation of such “micro-spheres… like individual bubbles in a mound of foam and are structured one layer over/under the other, without really being accessible to or separable from one another” (2004, p. 59).

As might by now be apparent from the discussion above, few spaces are seen to be riskier than treatment rooms in which coughing is itself part of treatment. Ellie (hospital consultant) explained that if room is used again and “they’ve coughed, there’s that potential for spread”. Some infections are seen to ‘linger’ longer than others. Nathan, another hospital consultant, spoke of work in the 1990s demonstrating the survival of the bacterium *pseudomonas* in treatment rooms for up to an hour after being vacated. He recounted a “massive outbreak” of resistant *pseudomonas*, an infection that went on to become epidemically established in the global CF community. Other infections, he explained, like mycobacterium abscessus can persist for 24 hrs or more after being coughed up. Ivor, a hospital microbiologist, highlighted the underlying uncertainties about the behaviour of pathogens once they leave the lungs and enter the spatio-temporal environment of the coughing body:

So if you’ve got a bug … you cough, where’s that bug going? Now for many of those bugs they may well float around in droplets, aerosolised particles … big ones might just, with gravity fall on the nearest surface, and they’ll dry out … most don’t survive terribly long on a dry clean surface… but quite a lot of them will float around… 40, 45 minutes for some organisms. It’s not terribly well studied, and we don’t know how far they travel and how long they exist for…

Many of our discussions return to the nature of air in treatment rooms and particularly the presence or absence of openable windows and their means of ventilation. Ivor referred back to the “Victorian, big open Nightingale wards” (Kisacky 2005). They may, he admits, have been “very cold and draughty” with patients “… wheeled outside in your bed on to a balcony”. But they were far more effective at changing the air than many contemporary hospital spaces. In opening a window, “you’re flushing the air in your room, in the way that you’re flushing a toilet, you’re flushing everything away… dispersing it”. Managing the risks of the coughing body in CF care routinely depends on being able to open the windows However, in the context of modern buildings, openable windows have often been ‘designed-out’ (Brown et al. 2020). Many respondents spoke of opening doors to treatment rooms to recirculate the air, although other participants in the study were just as anxious about open doors allowing infections to be carried on the air into adjoining corridors.

Other strategies involve, where possible, ‘resting’ treatment rooms between patients allowing time for pathogens to settle and desiccate. Here the absence of space is traded for a given span of time, although leaving treatment rooms inactive in busy clinical settings has proven difficult to justify to hospital management with an eye on optimising throughput. Containing the coughing CF body has come to depend on grouping patients (‘cohorting’) with the same pathogen, to be seen at about the same time, thus preventing different pathogens cross-infecting. This can extend to treating patients with more serious infections only after seeing those with less serious ones, thereby carefully ‘staggering’ patient appointments. Another spatio-temporal strategy is the ‘carousel’ where patients are ushered into a single treatment room to be visited by different specialists. All of these strategies are highly contingent on often erratic fluctuations in punctuality in the time critical choreography of respiratory care: “If they all turn up in their allocated time”, one hospital consultant explained, “the dance works beautifully … but of course they don’t”.

Treatment rooms are only one of a number of spaces to which coughing bodies become confined, or to which they retreat from the more open public spaces of the wider biopolitical *communitas* (Brown 2018). Coughing becomes caught between opposed and contradictory biomedical mandates and social obligations. The preservation of civic biopolitical order comes to depend on the withdrawal of the coughing body to zones of intimate personal privacy, especially those sanitarily associated with the hidden management of bodily fluids (see also Twigg 1999, 2000). As with treatment rooms, toilet facilities often tend to be small volume environments having cramped dimensions but crowded with sanitaryware. Indigo (patient) expressed acute anxieties in navigating shared toilets:

So I mean I always put my hand over my mouth when I cough, so straight away you’ve got germs on your hand… touch the toilet door… I cough so much…. If everyone else does that… the toilet flusher, the handrail, so you touch literally everything…. you’ve either got to not think about it, or you’d just go to a hyper extreme where we would all be levitating in plastic bubbles.

Wash facilities are also more likely to be windowless and poorly ventilated. And unlike treatment rooms, it is harder to control the simultaneous and quick succession of bodies passing through spaces in which intimate bodily processes take place. Yet these are also spaces to which people with CF often retreat to produce sputum in the absence of available treatment rooms. Echoing themes of stigma discussed above, some patients feel too conspicuous producing sputum in front of clinical staff and instead prefer to go to the bathroom.

Other confined ‘hot spots’ for the coughing body include lifts ubiquitously characteristic of deep density healthcare buildings. These are, again, enclosed airless spaces through which bodies are required to move simultaneously or in quick succession. One patient, Abbi (patient), put it “… the last thing you want is to get trapped in a lift with someone else with CF!”. To operate a lift also focusses anxieties on contact and hands: “… obviously you are touching the buttons … then you’re touching all the buttons on the inside. So that’s… the most likely source of [infection]… you cough into your hand, and you’ve touched the button, and then the other person touches the button”. In addition to concerns about lifts, Abbi expressed similar concerns about revolving or rotating doors forcing those passing through to momentarily share the same segment of space. At her hospital, the rotating doors, “a nightmare for cross-infection”, had been removed.

## Concluding discussion

Our fieldwork concluded in January 2020, just as the Covid-19 pandemic began to take hold across the world. Since then the coughing body has come to universal attention in a way unimaginable just a very short time ago. Our paper here documents the personal and clinical biopolitics of the coughing body in the lives of people with cystic fibrosis. However, it also reflects on practices that now have far wider significance in the context of a pandemic. As one sociological contribution on Covid-19 has recently put it, “our current experience of the pandemic is all about breath… risks in the simple act of breathing” (Will 2020; see also Brown et al. 2020).

Life with CF, as now in the Covid-19 pandemic, is also a highly charged symbolic and ritualised geometry of spatial positioning, and of gesturing and bodily etiquettes. Many of such practices are explored here, especially those associated with social distancing and bodily containment, but many other practices remain to be explored. At the time we undertook our research we could not, for example, have anticipated the extent to which masks and face coverings would become so enmeshed in the public scenography of pandemic life. Limited space precludes further exploration here; nevertheless, we have now become acutely aware of the highly contested nature of masks and ambiguity about their putative efficacy and their symbolism as outwardly visible signs of both pollution but also moral solidarity. Patients in our study spoke of the way masks made them feel uncomfortable, “you get a lot of stares” as one patient put it (Amy). Masks are also troubling objects of acute sensitivity in the sometimes-fragile relationship between patients and hospital staff. Rachel (physiotherapist) told us how her clinic had “agonised long and hard” about wearing masks, but “we’re treating humans” she went on. A hospital doctor however spoke of travelling through Asia and how “that really changed my mind… I always assumed that people wore masks because they didn’t want to catch things… but it’s the opposite; they don’t want to give things”. Again, this suggestion that masks can gesture civic responsibility is deeply prescient in the context of the Covid-19 world (see Wong 2020).

Covid-19 has brought to the surface ways of living and breathing that until recently lay hidden from view but which are detailed here, in the context of cystic fibrosis. This includes etiquettes of respiring, of having to contain one’s breath, of newly enclosing the open porosity of the body. Containing the cough echoes Mesman’s emphasis on ‘sterility as a product of spatial ordering’ (2009), now etched in markings on the street and in retail spaces directing people where to stand in line. Coughs have now also come to represent a form of interpersonal morality and ‘hygienic prudence’ (Lowton and Gabe 2006). Such ‘immunitary morality’ (Brown and Nettleton 2017) emplaces the coughing body in what Fox (1997) has called ‘circuits of hygiene’. We hope that our own work here with people who have cystic fibrosis, and with those who treat them, will form part of a newfound sociological engagement with a biopolitics of the air, of infections and transmission.
